# Assessing vaccinated persons’ intention to take the COVID-19 boosters using a combined theoretical framework: an online survey in Egypt

**DOI:** 10.1038/s41598-024-72093-9

**Published:** 2024-10-01

**Authors:** Maha El Tantawi, Amira H. Elwan, Reham Hassan, Nesreen Farouk Mohamed, Enas I. Elsheikh, Heba Ali Hassan, Sayed F. Abdelwahab

**Affiliations:** 1 Department of Pediatric Dentistry and Dental Public Health, Faculty of Dentistry, Alexandria University, Champolion, Alexandria, Egypt; 2 Department of Endodontics, Faculty of Dentistry, The Egyptian Russian University, Badr City, Egypt; 3 Department of Public Health, Community, Environmental and Occupational Medicine, Faculty of Medicine Port Said University, Port Said, Egypt; 4 Department of Pharmacognosy, Faculty of Pharmacy, Sohag University, Sohag, 82524 Egypt; 5 Department of Microbiology and Immunology, Faculty of Medicine, Minia University, Minia, 61511 Egypt; 6 Department of Endodontics, Faculty of Dentistry, Minia University, Minia, Egypt

**Keywords:** COVID-19 vaccines, Vaccination hesitancy, Health belief model, Theory of planned behaviour, Egypt, Preventive medicine, Epidemiology

## Abstract

Vaccines, like the Corona Virus Disease-2019 (COVID-19) vaccines, can control diseases, but vaccine hesitancy reduces their use. It is important to assess the intention to use COVID-19 vaccines boosters and the determinants of this intention to help in developing programs to promote the uptake of boosters. An online survey collected data from adults in Egypt between March and June 2022 using a questionnaire that assessed demographic characteristics, and constructs of the Theory of Planned Behaviour (TPB) and the Health Belief Model (HBM). The survey was uploaded to SurveyMonkey and the links were posted on social media platforms. Binary regression analysis was used and the dependent variable was intention to use boosters of COVID-19 vaccines. The independent variables were indicators of the HBM including perceived susceptibility to COVID-19 infection (medical history) and possibility of disease prevention (awareness of the availability of types of COVID-19 vaccines); and indicators of the TPB including attitude toward COVID-19 vaccines (that they are harmful, that they may lead to death and confidence in locally and foreign manufactured vaccines), perceived norms (the percentage of vaccinated persons in one’s circle) and perceived control over booster uptake (presence of government mandates for COVID-19 vaccination). The confounders were sociodemographic factors (age, sex, education, and place of residence). Complete responses were available from 1113 out of 1401 participants (79.4%), with mean (SD) age = 25 (9.5) years, of whom, 66.7% (n = 742) were females and 68.6% (n = 764) were university students. About 39.4% and 31.2% indicated that they would get or would definitely get the booster dose of the COVID-19 vaccines. In multiple regression, intention to use a booster dose was significantly related to not agreeing (AOR = 4.87, P < 0.001) or not agreeing at all (AOR = 8.46, P = 0.001) that vaccines are harmful and to having no confidence (AOR = 0.21, P < 0.001) or no confidence at all (AOR = 0.14, P < 0.001) in foreign-manufactured vaccines. Most university-educated Egyptians in the study intended to take the COVID-19 vaccine booster dose and this intention was associated with attitude toward the harm of the vaccine and confidence in foreign-manufactured vaccines. Awareness campaigns are needed to counteract misinformation and promote booster dose uptake.

## Introduction

The Corona Virus Disease-2019 (COVID-19) had severe effects on health and wellbeing, economy, and healthcare delivery systems^1,2^. Vaccines against the disease became available by the end of 2020^3^. Emergency use authorization was granted to two mRNA COVID-19 vaccines, the Pfizer-BioNTech and the Moderna COVID-19 vaccines and they protected against severe complications, reduced fatality rates, and deceased hospital admissions^4,5^. Millions of COVID-19 vaccine doses have been administered since December 2020. By 2021, it was clear that one dose of these vaccines would not provide adequate protection and that booster dose/s would be needed^6^. Several studies assessed booster dose acceptance and factors associated with intention to take boosters^7^. The percentage of persons willing to receive the COVID booster among the fully vaccinated was highest in Brazil (96%), Mexico (93%), China (90%), and lowest in Italy (66%) and Russia (62%)^8^. In the Middle East and North Africa (MENA), the intention to take the booster dose in different countries ranged from 45 to 78%^9–11^, and the overall estimate from several low and middle countries in the MENA region in another study was 26%^7^. Fear of complications and vaccine hesitancy were common among the populations in the region^12^.

Behaviour modification theories offer a framework to explain and address vaccination hesitancy. These theories include the health belief model (HBM) ^7^, the extended protection motivation theory ^13^, the theory of planned behaviour (TPB)^14^, and a combination of these theories^15^. According to the HBM, people engage in a preventive behaviour if they believe that they are vulnerable to a serious disease, that the disease can be prevented, and that they can manage the barriers against the preventive behaviour to prevent the disease^15^. On the other hand, the TPB^15^ explains the factors affecting the intention to adopt a behaviour and the intention, in turn, predicts behaviours. The TPB posits that intention depends on one’s attitude toward the behaviour, perceived control over the behaviour, and perceived norms regarding the behaviour. The HBM and the TPB provide a comprehensive framework to identify factors associated with vaccination^16^. Both theories explained Chinese parents’ decision to give a COVID-19 vaccine booster dose to their children and a study showed that attitude and behavioural control were significantly associated with parents’ intentions whereas subjective norms, perceived severity and susceptibility had no significant association with this intention^17^. Research also assessed the ability of constructs from the two theories to affect the uptake of the COVID-19 vaccine booster among adults in Italy and only subjective norms were directly associated with vaccination intention whereas perceived vulnerability and perceived disease severity were indirectly associated with vaccination intention through fear of COVID-19^18^. Thus, constructs from these two theories previously explained vaccine booster uptake in different settings and may, thus, be of value in explaining the same behaviour in Egypt.

In Egypt, there have been 516,023 confirmed cases of COVID-19 with 24,830 deaths until February 16th 2024^19^. Egypt ranked 7th after China, USA, Brazil, India, Italy, and France in case fatality rate due to COVID-19^20^. Also, Egypt had one of the lowest administration rates of the first dose of the COVID-19 vaccine and its boosters^21^. The low uptake of the vaccine was due to concerns about safety and side effects^22^. However, Egypt is one of the few middle-income countries that were able to locally produce COVID-19 vaccines^23^ and this was assumed to increase vaccine availability and boosters’ administration. A study assessed the intention of using the COVID-19 vaccine booster in Egypt and reported that 60.2% of participants were willing to receive a booster dose and 20.4% were reluctant^12^. The primary reasons for refusing the booster dose were uncertainty about safety and considering that the booster dose was not necessary. However, this study shed no light on the determinants of the intention to take a booster dose^12^. Another study assessed the opinions regarding COVID-19 vaccines in Egypt in 2021 and reported that 81.4% of participants were afraid of the vaccines, especially females and residents of rural areas^24^. In 2022, The Ministry of Health in Egypt conducted a national survey to assess the perception of COVID-19 vaccination and reported that only 41.8% were fully vaccinated. The study attributed incomplete vaccination to administrative issues such as crowded health facilities and registration problems but the determinants of booster dose uptake were not addressed^25^. More recently, it was reported that 47% of participants, of whom 29.3% were healthcare providers, refused to be vaccinated^26^, while 69% of university students accepted COVID-19 vaccination out of fear of getting infected^27^.

The World Health Organization (WHO) ended the international emergency because of the COVID-19 pandemic^28^. However, the study of vaccination and uptake of booster doses offer insights about the factors affecting the spread of the pandemic that are critical for controlling future pandemics. The international community is striving to ensure that preparedness activities are ongoing, to mobilize resources, and establish processes to address future scenarios. Research continues about the intention to take COVID-19 vaccine boosters and its determinants^29–32^. It is important to assess the impact of various determinants on the intention to receive a booster dose to design interventions that promote the uptake of vaccines and their boosters. This study assessed the intention to receive COVID-19 vaccines booster doses among vaccinated persons in Egypt and to identify factors associated with this intention based on a comprehensive theoretical framework including the HBM and the TPB. The study hypothesized that there would be no association between intention to receive a COVID-19 vaccine booster and the constructs of the HBM or the TPB.

## Methods

### Design

We used a cross-sectional online survey to collect data from participants living in Egypt between March and June 2022. This period followed an announcement by the Egyptian government of plans to export the locally produced COVID-19 vaccine to neighbouring African countries^33^. Ethical approval was obtained from the Research Ethics Committee, at the Faculty of Dentistry, Alexandria University (#0397-02/2022). The study was conducted in full accordance with the Helsinki declaration.

### Participants and sampling

We invited a convenience sample of people living in Egypt through social media and included participants if they were adults aged 18 years or older and had access to the survey through an electronic device and the internet. There were no exclusion criteria. Sample size was calculated using GPower to detect an odds ratio of at least 1.5 of intention to take the vaccine booster in relation to determinants in a two tailed test. We specified an alpha error = 5%, power = 80% with expected percentage of intention to take a booster dose = 60%^9,34^. To accommodate the independent variables with various distributions, the required sample size ranged from 215 to 844. Assuming non-response = 20%, 1013 ~ 1100 participants would be needed.

### Study questionnaire

We collected data using an anonymous, close-ended questionnaire based on a previous study^35^. The questionnaire included three sections (Appendix 1). The first section assessed participants’ demographic profile including age in years, sex (male or female), background (university student or other), and residence (the governorate in which the participant lived). The second section assessed medical history including reported history of chronic diseases (yes or no), history of COVID-19 infection (yes or no), COVID-19 vaccination history (yes or no) and perceived health (on a 5-point Likert scale ranging from very bad, bad, average, good, to very good). The third section assessed opinions toward COVID-19 vaccines including awareness of a number of COVID-19 vaccines available in Egypt (Oxford AstraZeneca, Moderna, Pfizer BionTech, Sinovac, Sinopharm, Sputnik and others)^36^, knowing that COVID-19 vaccine is mandatory in Egypt (yes, no, do not know), agreeing that vaccines are harmful (on a 5-point Likert scale ranging from not at all, no, neutral, yes, to yes completely), agreeing that vaccines may lead to death (yes, no, do not know), and confidence in locally- and foreign-manufactured vaccines (on a 4-point Likert scale including not at all, no, yes, and yes completely). This section also asked about the percentage of persons in the participants’ own circle who were vaccinated (none to 25%, more than 25 to 50%, more than 50 to 75% and more than 75 to 100%) and whether the participants intended to take the COVID-19 vaccine booster (on a 4-point Likert scale including definitely no, maybe no, maybe yes and definitely yes).

The study team developed the questions and response items in simple language to avoid confusion and elicit accurate responses. Two bilingual researchers, fluent in Arabic and English, independently translated the original questionnaire from English then back-translated the Arabic version to English and compared the two English versions to assess differences and edited to ensure accuracy and appropriateness to the local context^37^. We assessed content validity by asking six healthcare providers to rate the relevance of the items in the different sections and calculated the content validity ratio (CVR). The CVR was 0.86 which was greater than the recommended value^38^. We further piloted the questionnaire with persons from the general population including seven visitors to the outpatient clinic in Alexandria University hospital and they indicated that the questionnaire had good flow, the questions were easy to understand, and the options covered all scenarios.

A researcher uploaded the survey to SurveyMonkey. The survey included an introduction explaining the study purpose and inviting people to participate after affirming the confidentiality of responses, emphasizing voluntary participation, describing that participants can withdraw at any time and inviting them to communicate with the principal investigator if they had questions. Respondents had to click on a button to indicate consent to participate and procced to the survey. Participants could make only one submission per electronic device to avoid duplicate answers. No IPs or emails were collected to ensure confidentiality. The survey took 10–15 min to answer. The participants received no incentives to answer the survey.

### Data collection

The core team from Alexandria University reached out to researchers from various regions in Egypt to ensure geographic inclusion including the Delta, Upper Egypt, Suez Canal area, the Eastern area, the greater Cairo area, and Alexandria area. The extended study team members distributed the survey links to people in their networks using social media groups on Facebook, WhatsApp groups and Telegram for professionals, university students, and lay people. The survey links were posted in late March 2022 then reposted again in May then June to maximize response rates.

### Analysis

Data were cleaned and imported to SPSS version 23.0 (IBM Corp., Armonk, N.Y., USA). Only complete responses were used for analysis. We categorized the place of residence into 5 areas^39^: (i) greater Cairo (Cairo, Giza and Qalyubia), (ii) Alexandria area (Alexandria, Beheira and Matruh), (iii) Delta area (Dakahlia, Kafr El Sheikh, Gharbia, Menoufia and Damietta), (iv) Suez Canal area (Port Said, Ismailia, Suez, Sharqia, North Sinai and South Sinai), and (v) Upper Egypt (Beni Suef, Minya, Faiyum, Asyut, New Valley, Sohag, Qena, Luxor, Aswan and the Red Sea). We calculated the score for awareness of types of vaccines by adding the number of vaccine types the participants identified, ranging from zero (not aware of any type) to 7 (aware of all types). We selected the participants answering “yes” to the question about the history of COVID-19 vaccination for multiple binary logistic regression analysis.

#### Estimation model

The dependent variable was “intention to take a COVID-19 booster”, categorized into yes (definitely yes and maybe yes) and no (definitely no and maybe no). The independent variables were indicators of the HBM constructs: *perceived susceptibility to COVID-19 infection* including “history of chronic disease”, “perceived health” and “history of COVID-19 infection” and *possibility of preventing the disease* including “score of awareness of types of COVID-19 vaccines available in Egypt”. The third construct in the HBM, *ability to address barriers to the health behaviour* (booster uptake), was conceptualized as increased vaccine availability through local manufacture which applied at country level and, thus, we did not include it in the regression model since all participants were exposed to it. The indicators of the TPB constructs were *attitude toward COVID-19 vaccines* including “agreeing that vaccines were harmful”, “that they may lead to death” and “confidence in vaccines, local and foreign”; *perceived norms* including “percentage of vaccinated persons in the participant’s circle” and *perceived control over the behaviour* including “knowing about the presence of government mandates to be vaccinated against COVID-19”.

The confounders were socioeconomic indicators including “age”, “sex”, “background”, “place of residence” and “time in days since the beginning of the survey” as a quantitative variable because the survey was open for several months. The independent variables were introduced individually in a series of unadjusted models then together in a multiple adjusted model. We calculated the unadjusted and adjusted odds ratios (UORs and AORs), 95% confidence intervals (95% CIs) and p values as well as the unadjusted and adjusted estimated marginal effects of the logit model and displayed them for all subgroups and also included them in Appendix 2 for the full model. Significance level was set at 5%.

## Results

The study included 1113 complete responses out of 1401 collected responses (79.4%). Table 1 shows that the average (SD) age of participants was 25 (9.5) years, 66.7% (n = 742) were females, and 68.6% (n = 764) were university students. The greatest percentage of participants were from Alexandria region (31.9%; n = 355). Most participants had no chronic diseases, no history of COVID-19 infection and good/ very good perceived health. On average, they were aware of 3.7 types of COVID-19 vaccines available in Egypt out of 7 and indicated that COVID-19 vaccines were mandatory in Egypt (64.4%; n = 717). Also, 16% (n = 178) thought that vaccines may lead to death. A total of 43.4% had confidence or complete confidence in locally manufactured vaccines and 67.3% had confidence in foreign manufactured vaccines. Also, 76% thought that more than 50% of the people in their circle were vaccinated against COVID-19. In total, 70.6% (n = 785) expressed an intention to take the COVID-19 vaccine booster dose. Those who had already taken the COVID-19 vaccine were 1064 (95.6%) and regression analysis was restricted to them. No statistically significant differences existed between the whole sample and vaccinated persons in all factors (P > 0.05).

**Table 1:** Socioeconomic background, medical history, awareness, attitude toward vaccine, percentage of vaccinated acquaintances and intention to take COVID-19 vaccine booster dose.

Factors		All [n = 1113] N (%)	Vaccinated [n = 1064] N (%)
Age in years	Mean (SD)	25.0 (9.5)	24.8 (9.6)
Sex	Male	371 (33.3)	363 (34.1)
	Female	742 (66.7)	701 (65.9)
University student	Yes	764 (68.6)	764 (71.8)
	No	349 (31.5)	300 (28.2)
Place of residence	Greater Cairo area	147 (13.2)	134 (12.6)
	Alexandria area	355 (31.9)	342 (32.1)
	Delta area	114 (10.2)	110 (10.3)
	Suez Canal area	179 (16.1)	171 (16.1)
	Upper Egypt	318 (28.6)	307 (28.9)
Has chronic disease	Yes	55 (4.9)	52 (4.0)
	No	1058 (95.1)	1012 (95.1)
History of COVID-19 infection	Yes	419 (37.6)	393 (36.9)
	No	694 (62.4)	671 (63.1)
Perceived health	Very bad	2 (0.2)	2 (0.2)
	Bad	14 (1.3)	13 (1.2)
	Average	165 (14.8)	159 (14.9)
	Good	546 (49.1)	520 (48.9)
	Very good	386 (34.7)	370 (34.8)
Aware of types of COVID-19 vaccines available in Egypt (out of 7)	Mean (SD)	3.7 (1.8)	3.7 (1.8)
COVID-19 vaccination mandated in Egypt	Mandatory	717 (64.4)	686 (64.5)
	Elective	231 (20.8)	222 (20.9)
	Do not know	165 (14.8)	156 (14.7)
Thinking that vaccines lead to death	Yes	178 (16.0)	163 (15.3)
	No	496 (44.6)	483 (45.4)
	Do not know	439 (39.4)	418 (39.3)
Agreeing that vaccines are harmful	Not at all	71 (6.4)	71 (6.7)
	No	388 (34.9)	382 (35.9)
	Neutral	521 (46.8)	494 (46.4)
	Yes	85 (7.6)	75 (7.0)
	Yes, completely	48 (4.3)	42 (3.9)
Has confidence in locally manufactured vaccines	Not at all	158 (14.2)	142 (13.3)
	No	472 (42.4)	451 (42.4)
	Yes	447 (40.2)	437 (41.1)
	Yes, completely	36 (3.2)	34 (3.2)
Has confidence in foreign manufactured vaccines	Not at all	78 (6.8)	65 (6.1)
	No	286 (25.7)	267 (25.1)
	Yes	645 (58.0)	630 (59.2)
	Yes, completely	104 (9.3)	102 (9.6)
Percentage of people vaccinated in participant’s circle	0–25%	76 (6.8)	70 (6.6)
	> 25%-50%	191 (17.2)	178 (16.7)
	> 50%-75%	420 (37.7)	399 (37.5)
	> 75%-100%	426 (38.3)	417 (39.2)
Intention to take booster dose of COVID-19 vaccine	Definitely no	119 (10.7)	99 (9.3)
	Maybe no	209 (18.8)	194 (18.2)
	Maybe yes	438 (39.4)	428 (40.2)
	Definitely yes	347 (31.2)	343 (32.2)
Time in days since the survey was open		95	94

Table 2 shows that in unadjusted analysis, intention to take a booster dose of the COVID-19 vaccine was significantly associated with history of COVID-19 infection where participants with a history of COVID-19 infection had 31% higher odds of intending to use a booster dose than those without a history (P = 0.049). Not thinking that vaccine may lead to death was associated with 59% higher odds of intending to use a booster dose (P = 0.002) whereas not knowing whether vaccines lead to death was associated with 46% lower odds of intending to use a booster dose than thinking that vaccines lead to death. Agreeing that vaccines were not harmful at all, not harmful or being neutral about their harm was associated with 6.48% to 30.75% higher odds of intending to use a booster dose than completely agreeing that they were harmful (P < 0.001). Also, participants having no confidence at all in locally manufactured vaccines had 86% lower odds of intending to get a booster dose whereas participants with no confidence at all or no confidence in foreign manufactured vaccines had 97 to 88% lower odds of intending to take a booster dose than participants with complete confidence in locally and foreign manufactured vaccines respectively (P < 0.001). Participants with more than 25% of persons in their circle who were COVID-19 vaccinated had 118% to 205% higher odds of intending to take a booster dose than participants with 0–25% persons in their circle who were vaccinated (P < 0.05).

**Table 2:** Factors associated with intention to use a COVID-19 vaccine booster dose among vaccinated participants from Egypt (n = 1064) in multiple binary logistic regression (logit model) and estimated marginal effects.

Factors		UOR (95% CI)	Unadjusted estimated marginal effect (95% CI)	P value	AOR (95% CI)	Adjusted estimated marginal effect (95% CI)	P value
Chronic disease	Yes	0.99 (0.54, 1.77)	70.5 (67.7, 73.2)	0.95	1.21 (0.56, 2.70)	52.6 (25.7, 78.1)	0.67
	No^¶^	1.00	70.9 (57.7, 81.3)	-	1.00	48.5 (20.4, 77.6)	–
COVID-19 infection	Yes	1.31 (1.00, 1.70)	72.6 (69.2, 75.8)	0.049	1.16 (0.82, 1.63)	53.0 (25.7, 78.6)	0.26
	No^¶^	1.00	67.1 (62.4, 71.4)	–	1.00	48.1 (22.0, 75.3)	–
Perceived health	Very bad^¶^	1.00	50.0 (5.9, 94.1)	–	1.00	59.7 (0.07, 99.7)	–
	Bad	1.82 (0.09, 35.43)	64.3 (37.6, 84.3)	0.70	0.33 (0.002, 64.35)	34.1 (11.6, 67.1)	0.68
	Average	2.24 (0.14, 36.42)	69.1 (61.6, 75.7)	0.57	0.83 (0.005, 137.44)	55.7 (40.6, 69.8)	0.94
	Good	2.52 (0.16, 40.58)	71.6 (67.7, 75.2)	0.51	0.90 (0.005, 147.76)	54.5 (41.0, 67.4)	0.97
	Very good	2.33 (0.14, 37.53)	69.9 (65.2, 74.3)	0.55	0.67 (0.004, 111.12)	49.0 (35.6, 62.6)	0.88
Awareness of types of COVID-19 vaccines in Egypt		1.00 (0.93, 1.07)	–	0.91	0.97 (0.88, 1.06)	–	0.49
COVID-19 vaccines are mandated	Mandatory^¶^	1.00	68.2 (64.7, 71.4)	–	1.00	52.5 (25.4, 78.1)	–
	Elective	1.17 (0.74, 1.83)	76.2 (70.3, 81.2)	0.51	1.35 (0.74, 2.36)	52.2 (24.6, 78.5)	0.28
	Do not know	0.81 (0.56, 1.18)	73.2 (66.0, 79.4)	0.27	1.31 (0.82, 2.09)	46.9 (20.5, 75.2)	0.25
Vaccines lead to death	Yes^¶^	1.00	68.9 (64.5, 73.0)	–	1.00	50.3 (23.4, 77.0)	–
	No	1.59 (1.19, 2.12)	77.9 (74.1, 81.3)	0.002	1.05 (0.73, 1.50)	52.7 (25.2, 78.6)	0.78
	Do not know	0.54 (0.38, 0.78)	54.4 (47.1, 61.5)	0.001	0.91 (0.57, 1.46)	48.6 (22.0, 76.0)	0.70
Vaccines are harmful	Not at all	30.75 (10.97, 86.18)	90.1 (80.7, 95.2)	< 0.001	8.46 (2.45, 29.18)	76.9 (44.5, 93.2)	0.001
	No	21.73 (10.44, 45.27)	86.6 (82.8, 89.6)	< 0.001	4.87 (1.90, 12.47)	67.2 (38.0, 87.3)	0.001
	Neutral	6.48 (3.23, 13.01)	65.8 (61.7, 69.8)	< 0.001	2.44 (0.99, 5.98)	48.7 (22.4, 75.7)	0.05
	Yes	1.93 (0.86, 4.32)	36.5 (27.0, 47.2)	0.11	1.04 (0.38, 2.85)	29.8 (10.6, 60.3)	0.93
	Yes, completely^¶^	1.00	22.9 (13.2, 36.8)	–	1.00	28.8 (9.3, 61.6)	–
Confidence in local vaccines	Not at all	0.14 (0.06, 0.35)	37.3 (30.2, 45.1)	< 0.001	0.64 (0.20, 2.01)	33.8 (13.2, 63.1)	0.45
	No	0.45 (0.19, 1.06)	65.3 (60.8, 69.4)	0.07	1.69 (0.56, 5.11)	57.2 (29.0, 81.5)	0.36
	Yes	1.62 (0.68, 3.87)	87.0 (83.6, 89.8)	0.28	2.98 (1.00, 8.81)	68.6 (39.9, 87.8)	0.05
	Yes, completely^¶^	1.00	80.6 (64.5, 90.4)	–	1.00	42.2 (13.9, 76.8)	–
Confidence in foreign vaccines	Not at all	0.03 (0.01, 0.07)	20.5 (13.0, 30.9)	< 0.001	0.14 (0.05, 0.39)	24.9 (8.2, 55.3)	< 0.001
	No	0.12 (0.06, 0.22)	49.7 (43.9, 55.4)	< 0.001	0.21 (0.09, 0.49)	35.6 (14.1, 65.2)	< 0.001
	Yes	0.57 (0.29, 1.10)	82.8 (79.7, 85.5)	0.09	0.66 (0.31, 1.43)	66.2 (37.4, 86.6)	0.30
	Yes, completely^¶^	1.00	89.4 (81.9, 94.0)	–	1.00	75.1 (44.4, 91.9)	–
Percent of persons vaccinated in one’s circle	0–25%^¶^	1.00	51.3 (40.2, 62.3)	–	1.00	41.1 (16.4, 71.1)	–
	> 25%-50%	2.18 (1.26, 3.75)	69.6 (62.7, 75.7)	0.005	1.66 (0.83, 3.34)	57.2 (28.1, 82.0)	0.15
	> 50%-75%	2.07 (1.26, 3.38)	68.6 (64.0, 72.8)	0.004	1.28 (0.66, 2.43)	49.3 (22.8, 76.3)	0.45
	> 75%-100%	3.05 (1.85, 5.04)	76.3 (72.0, 80.1)	< 0.001	1.56 (0.81, 2.98)	54.6 (26.7, 79.9)	0.18
Time in days since the survey was open		0.98 (0.97, 1.02)	–	0.95	1.01 (0.98, 1.03)	–	0.99

The multiple model was adjusted for age, sex, place of residence and whether participant was a university student.

UOR unadjusted odds ratio, AOR adjusted odds ratio, CI confidence interval.

^¶^Reference category.

In multiple regression, intending to take a booster dose was significantly associated with not agreeing (P < 0.001) or not agreeing at all (P = 0.001) that vaccines were harmful. Those who did not agree that vaccines were harmful had 387% higher odds and those who did not agree at all that vaccines were harmful had 746% higher odds of intending to take a booster dose than those who completely agreed that vaccines were harmful. There was a significant association between intending to take a booster dose and having no confidence or no confidence at all (P < 0.001) in foreign manufactured vaccines. Those with no confidence had 79% lower odds and those with no confidence at all had 86% lower odds of taking a booster dose than those with complete confidence in foreign manufactured vaccines.

Based on the regression estimates in the logit model, the logit equation was.

log(p/1-p) = -0.668 + 0.017*χ1 – 0.115*χ2 – 0.505*χ3 + 0.666*χ4-1 +  + 0.623*χ4-2 + 0.184*χ4-3 + 0.0.815*χ4-4 + 0.166*χ5 + 0.194*χ6 + 0.433*χ7-1 – 0.622*χ7-2 + 0.267*χ7-3 + 0.220*χ7-4 – 0.032*χ8 – 0.222*χ9-1 – 0.013*χ9-2 + 0.066*χ10-1 + 0.164*χ10-2 + 2.105*χ11-1 + 1.622*χ11-2 + 0.852*χ11-3 + 0.046*χ11-4 – 0.361*χ12-1 + 0.604*χ12-2 + 1.093*χ12-3 – 2.207*χ13-1 – 1.697*χ13-2 – 0.431*χ13-3 – 0.547*χ14-1 + 0.105*χ14-2 – 0.212*χ14-3 + 0.011*χ15.

where p is the probability of intending to take a booster dose of the COVID-19 vaccine, χ1 to χ15 are the variables listed in Table 1 and χ4-1 to χ4-4,….. are the categories of the variable χ4 and so on.

The estimated marginal effects in Table 2 show the same directions of relationships as the UORs and the AORs. In unadjusted analysis, the greatest differences between subgroups in intention to use a booster dose marginal effects were between participants who agreed that vaccines were not at all harmful and those who completely agreed they were harmful (90.1 and 22.9%) which were reduced in adjusted analysis to 76.9 and 28.8% respectively and the difference between participants who had no confidence at all in foreign manufactured vaccines and those who had complete confidence in them (20.5 and 89.4%) which were reduced in adjusted analysis to 24.9 and 75.1% respectively 

## Discussion

The study showed that in a group of vaccinated, university-educated students living in Egypt, intention to take a COVID-19 vaccine booster dose differed based on the attitude toward COVID-19 vaccine harm and confidence in foreign manufactured vaccines. An attitude considering the vaccine safe was associated with higher odds of intention to take a booster. Participants with no confidence in foreign manufactured vaccines had lower odds of intending to take a booster dose. Attitude toward COVID-19 vaccines seemed to have the greatest impact on intention to take a COVID-19 booster dose whereas perceived control, social norms and perceived susceptibility may have less importance. The study suggests that educational programs promoting COVID-19 vaccination should focus on changing the attitude of educated persons toward COVID-19 vaccines. The findings partly support the study hypothesis.

The present study has some limitations. The study is limited by its cross-sectional design which does not prove causality and can only show associations. Online surveys, like the one used in the present study and in most COVID-19 studies, are likely to have some selection bias because it is not possible to use random samples when the survey is posted online. The convenience sampling, which was used instead, meant that some subgroups had a higher probability of being included in the sample than others. In the present study, the sample mostly consisted of university students whereas statistics show that about 30% of Egyptians are illiterate^40^. Thus, the sample was skewed to university-educated persons. Also, the questionnaire was distributed through an online platform whereas the internet penetration rate in Egypt was 71.9% in 2022^41^. Thus, specific groups in the population were under-represented because they had less access to the Internet. Therefore, although most regions in Egypt were represented, the findings may not apply to the general Egyptian population. We assessed participants’ income level, but most participants were university students who were still dependent on their parents for financial support. They reported the allowance they received from their parents as income and this would underestimate their socio-economic level. It is important to consider the sample profile when generalizing the study findings. The results are applicable to young, university-educated students with similar cultural backgrounds. Older populations with less education may have a lower level of intention to receive booster doses of the COVID-19 vaccine. Nevertheless, the study has several strengths including the large sample size, the wide geographic coverage at national level and the use of behavioural change theories to explain the health behaviour.

The study had several important findings. First, about three quarters of the participants, who were mainly university students, reported an intention to take a COVID-19 vaccine booster dose similar to a previous study^12^. Despite this, the percentage of people receiving the COVID-19 vaccine and its booster doses in Egypt remains one of the lowest worldwide^21,25^. It is important to address the obstacles preventing the intention to take the COVID-19 vaccine and its booster dose from becoming actual behaviors and to monitor the reduction in this gap as time passes and misinformation about the COVID-19 vaccines dispel. It is also important to acknowledge the importance of vaccine hesitancy beyond the COVID-19 pandemic context and how it impacts the spread of vaccine-preventable diseases worldwide^42^ which adds to the importance of the present study.

Second, people with a history of COVID-19 infection had 30% higher odds of expressing an intention to take a booster dose in unadjusted analysis. The physical and mental impact of COVID-19 infection^43^ may increase perceived vulnerability causing affected individuals to seek the protection offered by COVID-19 vaccine booster doses^44^. Our findings disagree with research^45^ showing that previous COVID-19 infection was associated with disinclination to receive boosters. The difference between this and our study may be attributed to the time of data collection. Earlier research might have been conducted at a stage in the pandemic when people believed that COVID-19 infection conferred lifelong immunity with no need for vaccination among those who have already been infected^46^.

Third, the awareness that vaccination was mandatory seemed not to be related to perceived control of the uptake of booster doses in the present study and was not significantly associated with the intention to take a booster dose. Vaccination mandates have faced opposition, with citizens demonstrating against them in several countries^47^. Research^48^ showed that mandates were likely to increase vaccination in countries where vaccination rates were initially modest, if vaccine supplies were available, in subgroups likely to face penalties if they did not follow the mandates such as visitors not allowed inside public facilities without proof of vaccination. These conditions applied to the participants in the present study. However, the question we used in the survey was about mandated COVID-19 vaccination not its booster dose which was not specifically mandated in Egypt.

Fourth, perceiving that more persons were vaccinated in one’s circle was associated with 118% to 205% higher odds of intention to take a booster dose in unadjusted analysis. In our study, 76% of participants thought that at least half the people in their circles were vaccinated. This perception of high vaccination rate may be because of the governmental decree preventing non vaccinated persons from entering public facilities in Egypt^49^. Campaigns in universities resulted in higher vaccination rates than the general population^50^. It is important to note that the intention to take a booster dose was not associated with being a student or with awareness that vaccination was mandated but was associated with perceiving vaccination to be the norm. This emphasizes the importance of perceived norms in shaping one’s intentions compared to rules or group membership per se. However, the observed association was not significant in the adjusted analysis when attitude was included. The complex interplay between the individual and the community, attitude and norms, intention and actual behavior needs exploration in future studies.

Fifth, a positive attitude toward the COVID-19 vaccine harmlessness was associated with 387% to 746% significantly higher odds of intention to take a booster dose in agreement with previous studies in other countries^51,52^ and in Egypt^53^. There was also greater confidence in foreign than locally manufactured vaccines, which disagrees with research showing bias to locally produced vaccines^54^. This difference may be because at the time of the study, Egypt had just started to locally manufacture the Sinovac vaccine^23^, and not all participants were aware of this development. Another reason may be related to the low confidence in the Sinovac vaccine itself^55^. However, the WHO selected Egypt and five other countries in Africa to produce the Pfizer-BioNTech^®^ and the Moderna^®^ vaccines^56^. Confidence in these vaccines may differ from that in the Sinovac vaccine and this needs to be assessed in future studies.

Sixth, in this study, attitude was significantly associated with intention to take a COVID-19 vaccine booster dose in adjusted regression where participants with no confidence in foreign manufactured vaccines had 79–86% lower odds of intending to take a booster dose. This finding agrees with studies from Germany^57^ and China^58^ showing that attitude toward the vaccine was associated with vaccination intention. By contrast, studies from Ethiopia^59^, India^60^, Pakistan^61^, and the United States^62^ reported associations between attitude, norms and control with vaccination intention. Other studies^63–66^ showed that both the TPB and the HBM constructs were associated with vaccination indicating the complexity of booster uptake as a behavior and possible specificity of country-level determinants.

## Conclusion

This study showed that most young and university-educated Egyptians participating in the study intended to take a COVID-19 vaccine booster dose and that this intention was associated with attitude toward vaccine harm and confidence in foreign vaccines. Even among young and university-educated individuals, unfavorable attitudes may negatively impact full vaccination and, therefore, the control of pandemics. Awareness campaigns are needed to overcome the negative attitudes and disinformation that discourage people from taking booster doses.

## Supplementary Information

Supplementary Information.

## Data Availability

The data used and/or analyzed during the current study is available from the corresponding author on reasonable request.

## References

[1] Haleem, A., Javaid, M. & Vaishya, R. Effects of COVID-19 pandemic in daily life. *Curr. Med. Res. Pract.* **10**, 78–79. 10.1016/j.cmrp.2020.03.011 (2020). 32292804 10.1016/j.cmrp.2020.03.011 PMC7147210

[2] World Health Organization. The impact of COVID-19 on global health goals. Accessed November 2, 2022 at: https://www.who.int/news-room/spotlight/the-impact-of-covid-19-on-global-health-goals (2021).

[3] The College of Physicians of Philadelphia. Coronavirus | History of Vaccines. Accessed November 2, 2022 at https://historyofvaccines.org/diseases/coronavirus

[4] Food and Drug Administration. FDA Takes Key Action in Fight Against COVID-19 By Issuing Emergency Use Authorization for First COVID-19 Vaccine. Accessed November 2, 2022 at: https://www.fda.gov/news-events/press-announcements/fda-takes-key-action-fight-against-covid-19-issuing-emergency-use-authorization-first-covid-19 (2020).

[5] Food and Drug Administration. FDA Takes Additional Action in Fight Against COVID-19 By Issuing Emergency Use Authorization for Second COVID-19 Vaccine. Accessed November 2, 2022 at: https://www.fda.gov/news-events/press-announcements/fda-takes-additional-action-fight-against-covid-19-issuing-emergency-use-authorization-second-covid (2020).

[6] Marra, A. R. *et al.* The short-term effectiveness of coronavirus disease 2019 (COVID-19) vaccines among healthcare workers: A systematic literature review and meta-analysis. *Antimicrob. Steward. Healthc. Epidemiol.*10.1017/ASH.2021.195 (2021). 36168453 10.1017/ash.2021.195 PMC9495770

[7] Ghazy, R. M. *et al.* Acceptance of COVID-19 vaccine booster doses using the health belief model: A cross-sectional study in low-middle- and high-income countries of the east mediterranean region. *Int. J. Environ. Res. Public Health* **19**, 12136. 10.3390/IJERPH191912136 (2022). 36231447 10.3390/ijerph191912136 PMC9566578

[8] Ipsos. Most adults vaccinated against COVID-19 in all 13 countries surveyed intend to get a booster shot. Accessed December 1, 2023 at: https://www.ipsos.com/en/global-attitudes-covid-19-vaccine-booster-shots (2021).

[9] Al-Qerem, W. *et al.* Willingness of the Jordanian population to receive a COVID-19 booster dose: A cross-sectional study. *Vaccines*10.3390/VACCINES10030410 (2022). 35335042 10.3390/vaccines10030410 PMC8950968

[10] Al-Qerem, W. *et al.* Knowledge, attitudes, and practices of adult Iraqi population towards COVID-19 booster dose: A cross-sectional study. *Patient Prefer. Adherence* **16**, 1525–1537. 10.2147/PPA.S370124 (2022). 35769339 10.2147/PPA.S370124 PMC9236163

[11] Moeed, A. *et al.* Willingness and perceptions regarding COVID-19 vaccine booster dose in Pakistani vaccinated population: A cross-sectional survey. *Front. public health.*10.3389/FPUBH.2022.911518 (2022). 35844859 10.3389/fpubh.2022.911518 PMC9279681

[12] Abouzid, M. *et al.* Attitudes toward receiving COVID-19 booster dose in the Middle East and North Africa (MENA) region: A cross-sectional study of 3041 fully vaccinated participants. *Vaccines* **10**, 1270. 10.3390/VACCINES10081270 (2022). 36016158 10.3390/vaccines10081270 PMC9414713

[13] Liu, M. *et al.* Using an extended protection motivation theory to explain vaccine hesitancy: A cross-sectional study among Chinese adults. *Human vaccin. immunother.*10.1080/21645515.2022.2026136 (2022). 10.1080/21645515.2022.2026136 PMC8993063 35103578

[14] Lee, K. W. *et al.* COVID-19 vaccine hesitancy and its associated factors in Malaysia. *PLoS ONE* **17**, e0266925. 10.1371/journal.pone.0266925 (2022). 36048822 10.1371/journal.pone.0266925 PMC9436036

[15] Romate, J. *et al.* Using the integrative model of behavioural prediction to understand COVID-19 vaccine hesitancy behaviour. *Sci. Rep.*10.1038/S41598-022-12466-0 (2022). 35661762 10.1038/s41598-022-12466-0 PMC9166190

[16] Short, M. B. *et al.* Understanding factors associated with intent to receive the COVID-19 vaccine. *Fam. Syst. Health* **40**, 160–170. 10.1037/FSH0000664 (2022). 35666894 10.1037/fsh0000664

[17] Zhou, M. *et al.* Behavioral intention and its predictors toward COVID-19 booster vaccination among Chinese parents: Applying two behavioral theories. *Int. J. Environ. Res. Public Health*10.3390/IJERPH19127520 (2022). 35742770 10.3390/ijerph19127520 PMC9224228

[18] Barattucci, M. *et al.* Trust in science as a possible mediator between different antecedents and COVID-19 booster vaccination intention: An integration of health belief model (HBM) and theory of planned behavior (TPB). *Vaccines*10.3390/VACCINES10071099 (2022). 35891265 10.3390/vaccines10071099 PMC9320855

[19] World Health Organization. Confirmed cases of COVID-19 in Egypt. Accessed December 1, 2023 at: https://covid19.who.int/region/emro/country/eg (2023).

[20] Saied, A. A., Metwally, A. A., Madkhali, N. A. B., Haque, S. & Dhama, K. Egypt’s COVID-19 recent happenings and perspectives: A mini-review. *Front. Public Health* **9**, 696082. 10.3389/fpubh.2021.696082 (2021). 34485226 10.3389/fpubh.2021.696082 PMC8415352

[21] Our World in Data. Coronavirus Pandemic (COVID-19). Accessed November 2, 2022 at: https://ourworldindata.org/coronavirus (2020).

[22] Dadras, O. *et al.* Public acceptability of COVID-19 vaccines and its predictors in Middle Eastern/North African (MENA) countries: A systematic review. *Human Vaccin. Immunother.*10.1080/21645515.2022.2043719 (2022). 10.1080/21645515.2022.2043719 PMC9196809 35318872

[23] Egypt Today. Egypt aims for cooperation with Cuba on vaccine production. Accessed November 2, 2022 at: https://www.egypttoday.com/Article/1/114996/Egypt-aims-for-cooperation-with-Cuba-on-vaccine-production (2022).

[24] Elareed, H. *et al.* Public opinion towards COVID-19 vaccination in Egypt. *Egypt. J. Community Med.* **41**, 27–35. 10.21608/ejcm.2022.135572.1214 (2023).

[25] Kandeel, A. *et al.* COVID-19 vaccination coverage in Egypt: a large-scale national survey—to help achieving vaccination target, March-May, 2022. *BMC Public Health* **23**, 397. 10.1186/s12889-023-15283-w (2023). 36849954 10.1186/s12889-023-15283-w PMC9969364

[26] Shawki, M. A. *et al.* COVID-19 vaccine hesitancy in Egypt: a cross-sectional study. *J. Infect. Dev. Ctries.* **17**, 1188–1198. 10.3855/jidc.17794 (2023). 37824346 10.3855/jidc.17794

[27] Tharwat, S., Saad, A. M., Nassar, M. K. & Nassar, D. K. Acceptance and hesitancy to receive COVID-19 vaccine among university students in Egypt: A nationwide survey. *Trop. Med. Health* **51**, 16. 10.1186/s41182-023-00509-9 (2023). 36895057 10.1186/s41182-023-00509-9 PMC9995735

[28] Reuters. WHO declares end to COVID global health emergency. Accessed June 27, 2023 at: https://www.reuters.com/business/healthcare-pharmaceuticals/covid-is-no-longer-global-health-emergency-who-2023-05-05/ (2023).

[29] Huang, P. C. *et al.* Expanding Protection Motivation Theory to explain vaccine uptake among United Kingdom and Taiwan populations. *Hum. Vaccin. Immunother.* **19**, 2211319. 10.1080/21645515.2023.2211319 (2023). 37212327 10.1080/21645515.2023.2211319 PMC10294722

[30] Huang, M. *et al.* COVID-19 vaccine booster dose hesitancy among key groups: A cross-sectional study. *Hum. Vaccin. Immunother.* **19**, 2166323. 10.1080/21645515.2023.2166323 (2023). 36951564 10.1080/21645515.2023.2166323 PMC10072063

[31] Hansen, B. T., Labberton, A. S., Kour, P. & Kraft, K. B. Coverage of primary and booster vaccination against COVID-19 by socioeconomic level: A nationwide cross-sectional registry study. *Hum. Vaccin. Immunother.* **19**, 2188857. 10.1080/21645515.2023.2188857 (2023). 36941785 10.1080/21645515.2023.2188857 PMC10072069

[32] Qin, C. *et al.* Assessing acceptability of the fourth dose against COVID-19 among Chinese adults: A population-based survey. *Hum. Vaccin. Immunother.* **19**, 2186108. 10.1080/21645515.2023.2186108 (2023). 36892289 10.1080/21645515.2023.2186108 PMC10026929

[33] Arab News. Egypt produces 30m doses of Vaccera-Sinovac vaccine. Accessed June 27, 2023 at: https://www.arabnews.com/node/2029116/middle-east (2023).

[34] Lounis, M. *et al.* COVID-19 vaccine booster hesitancy (vbh) and its drivers in Algeria: National cross-sectional survey-based study. *Vaccines.*10.3390/VACCINES10040621 (2022). 35455371 10.3390/vaccines10040621 PMC9031698

[35] Chu, D. T. *et al.* Willingness to receive COVID-19 vaccine booster doses for adults and their children in Vietnam. *J. Hum. Behav. Soc. Environ.*10.1080/10911359.2022.2046235 (2022).

[36] COVID19 Vaccine Tracker. Egypt. Accessed November 2, 2022 at: https://covid19.trackvaccines.org/country/egypt/ (2022).

[37] World Health Organization. WHODAS 2.0 TRANSLATION PACKAGE (VERSION 1.0) Accessed December 1, 2023 at: https://terrance.who.int/mediacentre/data/WHODAS/Guidelines/WHODAS%202.0%20Translation%20guidelines.pdf

[38] Yusoff, M. S. B. ABC of content validation and content validity index calculation. *Edu. Med. J.* **11**, 49–54. 10.21315/eimj2019.11.2.6 (2019).

[39] Wikipedia. List of Egypt's governorates. Accessed November 2, 2022 at: https://ar.wikipedia.org/wiki/قائمة_محافظات_مصر#المحافظات_حسب_الأقليم (2022).

[40] TheGlobalEconomy.com. Egypt economic indicators. Accessed November 2, 2022 at: https://www.theglobaleconomy.com/Egypt/ (2022).

[41] Data Reportal. DIGITAL 2022: EGYPT. Accessed December 1, 2023 at: https://datareportal.com/reports/digital-2022-egypt#:~:text=There%20were%2075.66%20million%20internet,percent)%20between%202021%20and%202022 (2022).

[42] Larson, H. J., Jarrett, C., Eckersberger, E., Smith, D. M. D. & Paterson, P. Understanding vaccine hesitancy around vaccines and vaccination from a global perspective: A systematic review of published literature, 2007–2012. *Vaccine* **32**, 2150–2159. 10.1016/j.vaccine.2014.01.081 (2014). 24598724 10.1016/j.vaccine.2014.01.081

[43] Dai, J. *et al.* The Influence of COVID-19 pandemic on physical health-psychological health, physical activity, and overall well-being: The mediating role of emotional regulation. *Front. Psychol.* **12**, 3005. 10.3389/FPSYG.2021.667461/BIBTEX (2021). 10.3389/fpsyg.2021.667461 PMC8415626 34484032

[44] Mertens, G., Lodder, P., Smeets, T. & Duijndam, S. Fear of COVID-19 predicts vaccination willingness 14 months later. *J Anxiety Disord* **88**, 102574. 10.1016/j.janxdis.2022.102574 (2022). 35512598 10.1016/j.janxdis.2022.102574 PMC9047433

[45] Abdelmoneim, S. A. *et al.* COVID-19 vaccine booster dose acceptance: Systematic review and meta-analysis. *Trop. Med. Infect. Dis.*10.3390/tropicalmed7100298 (2022). 36288039 10.3390/tropicalmed7100298 PMC9611447

[46] World Health Organization. Interim statement on hybrid immunity and increasing population seroprevalence rates. Accessed November 2, 2022 at: https://www.who.int/news/item/01-06-2022-interim-statement-on-hybrid-immunity-and-increasing-population-seroprevalence-rates (2022).

[47] Drew, L. Did COVID vaccine mandates work? What the data say. *Nature* **607**, 22–25. 10.1038/D41586-022-01827-4 (2022). 35794272 10.1038/d41586-022-01827-4

[48] Mills, M. C. & Rüttenauer, T. The effect of mandatory COVID-19 certificates on vaccine uptake: Synthetic-control modelling of six countries. *Lancet Public Health* **7**, e15–e22. 10.1016/S2468-2667(21)00273-5 (2022). 34914925 10.1016/S2468-2667(21)00273-5 PMC8668192

[49] Egypt Independent. Egypt enforces move to ban all unvaccinated citizens from government offices. Accessed November 9, 2022 at: https://egyptindependent.com/egypt-enforces-move-to-ban-all-unvaccinated-citizens-from-government-offices/ (2021).

[50] Egypt Speeds Up Vaccination for University Students | Asharq AL-awsat.

[51] Rahman, M. M. *et al.* Knowledge, attitude, and hesitancy towards COVID-19 vaccine among university students of Bangladesh. *PloS ONE* **17**, e0270684 (2022). 35759475 10.1371/journal.pone.0270684 PMC9236250

[52] Sonmezer, M. C. *et al.* Knowledge, attitudes, and perception towards COVID-19 vaccination among the adult population: A cross-sectional study in Turkey. *Vaccines* **10**, 278. 10.3390/VACCINES10020278/S1 (2022). 35214736 10.3390/vaccines10020278 PMC8877322

[53] Salem, G. M. *et al.* Acceptance rate of COVID-19 vaccination and its predictors in Egypt: An online survey. *J. Infect. Dev. Ctries.* **16**, 993–1000. 10.3855/JIDC.15603 (2022). 35797293 10.3855/jidc.15603

[54] Barceló, J. *et al.* Vaccine nationalism among the public: A cross-country experimental evidence of own-country bias towards COVID-19 vaccination. *Soc. Sci. Med.*10.1016/J.SOCSCIMED.2022.115278 (2022). 35994879 10.1016/j.socscimed.2022.115278 PMC9376148

[55] The Cable News Network. Sinovac: Confidence in Chinese vaccines has taken a hit. But as coronavirus cases grow, some countries are still pushing ahead | CNN. Accessed November 2 2022 at: https://edition.cnn.com/2021/01/17/asia/sinovac-vaccine-asia-efficacy-intl-hnk/index.html (2021).

[56] World Health Organization. WHO announces first technology recipients of mRNA vaccine hub with strong support from African and European partners. Accessed November 14, 2022 at: https://www.who.int/news/item/18-02-2022-who-announces-first-technology-recipients-of-mrna-vaccine-hub-with-strong-support-from-african-and-european-partners (2022).

[57] Seddig, D. *et al.* Correlates of COVID-19 vaccination intentions: Attitudes, institutional trust, fear, conspiracy beliefs, and vaccine skepticism. *Soc. Sci. Med.*10.1016/J.SOCSCIMED.2022.114981 (2022). 35512613 10.1016/j.socscimed.2022.114981 PMC9017059

[58] Fan, C. W. *et al.* Extended theory of planned behavior in explaining the intention to COVID-19 vaccination uptake among mainland Chinese university students: an online survey study. *Hum. Vaccin. Immunother.* **17**, 3413–3420. 10.1080/21645515.2021.1933687 (2021). 34170792 10.1080/21645515.2021.1933687 PMC8437493

[59] Asmare, G. *et al.* Behavioral intention and its predictors toward COVID-19 vaccination among people most at risk of exposure in Ethiopia: applying the theory of planned behavior model. *Hum. Vaccin. Immunother.* **17**, 4838–4845. 10.1080/21645515.2021.2011651 (2021). 35213947 10.1080/21645515.2021.2011651 PMC8903977

[60] Husain, F. *et al.* Intention to get COVID-19 vaccines: Exploring the role of attitudes, subjective norms, perceived behavioral control, belief in COVID-19 misinformation, and vaccine confidence in Northern India. *Hum. Vaccin. Immunother.* **17**, 3941–3953. 10.1080/21645515.2021.1967039 (2021). 34546837 10.1080/21645515.2021.1967039 PMC8828102

[61] Ullah, I. *et al.* Factors affecting Pakistani young adults’ intentions to uptake COVID-19 vaccination: An extension of the theory of planned behavior. *Brain Behav.*10.1002/BRB3.2370 (2021). 34543522 10.1002/brb3.2370 PMC8613438

[62] Hayashi, Y. *et al.* Predicting intention to take a COVID-19 vaccine in the United States: Application and extension of theory of planned behavior. *Am. J. Health Promot.* **36**, 710–713. 10.1177/08901171211062584 (2022). 35041541 10.1177/08901171211062584 PMC9014362

[63] Hossain, M. B. *et al.* Health belief model, theory of planned behavior, or psychological antecedents: What Predicts COVID-19 vaccine hesitancy better among the bangladeshi adults?. *Front. Public Health*10.3389/FPUBH.2021.711066 (2021). 34490193 10.3389/fpubh.2021.711066 PMC8418098

[64] Berg, M. B. & Lin, L. Predictors of COVID-19 vaccine intentions in the United States: The role of psychosocial health constructs and demographic factors. *Transl. Behav. Med.* **11**, 1782–1788. 10.1093/TBM/IBAB102 (2021). 34293163 10.1093/tbm/ibab102 PMC8344533

[65] Shmueli, L. Predicting intention to receive COVID-19 vaccine among the general population using the health belief model and the theory of planned behavior model. *BMC Public Health* **21**, 804. 10.1186/S12889-021-10816-7 (2021). 33902501 10.1186/s12889-021-10816-7 PMC8075011

[66] Guidry, J. P. D. *et al.* Willingness to get the COVID-19 vaccine with and without emergency use authorization. *Am. J. Infect. Control* **49**, 137–142. 10.1016/J.AJIC.2020.11.018 (2021). 33227323 10.1016/j.ajic.2020.11.018 PMC7677682

