# Supplementary material for: Assessing vaccinated persons’ intention to take the COVID-19 boosters using a combined theoretical framework: an online survey in Egypt
Source: Sci Rep. 2024 Oct 1;14:22795. doi: 10.1038/s41598-024-72093-9 (PMC11445433; doi:10.1038/s41598-024-72093-9)
Supplement: Supplementary file 1 — Supplementary Information. [file 41598_2024_72093_MOESM1_ESM.pdf]

Assessing vaccinated persons' intention to take the COVID-19 boosters using a combined theoretical framework: an online survey in Egypt

Maha El Tantawi, Amira Elwan, Reham Hassan, Nesreen Farouk Mohamed, Enas I. Elsheikh, Heba Ali Hassan, Sayed F. Abdelwahab

Appendix 1

Research on the willingness to take the COVID-19 vaccine
استبيان عن الاستعداد لتلقي لقاح الكورونا

Section 1: General information<br>القسم الأول: معلومات عامة		
1	Age السن	________________________________years old
2	Gender النوع	[1] Maleذكر<br>[2] Femaleأنثى
3	Highest education level أعلى مستوى تعليم حصلت عليه	[1] Primary school and below الشهادة الابتدائية أو أقل منها<br>[2] Secondary schoolالشهادة الاعدادية<br>[3] High schoolالشهادة الثانوية<br>[4] University and above الشهادة الجامعية فأعلى
4	Occupation الوظيفة	[1] Industrial workers عامل بناء<br>[2] Farmers فلاح - مزارع<br>[3] Self-employed عمل حر<br>[4] Officials and employees موظف<br>[5] Students طالب<br>[6] Businessman رجل أعمال<br>[7] Retiredمتقاعد – على المعاش<br>[8] Othersأخرى
5	Average monthly income ما هو متوسط دخلك الشهري	[1] ≤ 2700 جنيه شهريا<br>[2] > 2700 جنيه شهريا
6	Current residence: governorate محل السكن (المحافظة)	___ ______________________________________
Section 2: General health<br>القسم الثاني: الصحة العامة		
7	Ever have been infected with COVID-19?هل أصبت بالكورونا من قبل؟	[1] Yes نعم<br>[2] No لا
8	Do you have an existing chronic disease such as cancer, cardiovascular disease, diabetes, etc<br>هل تعاني من مرض مزمن ؟ كالسرطان، أمراض القلب و الشرايين التاجية، او السكري و خلافه؟	[1] Yes نعم<br>[2] No لا
9	How do you rate your overall health كيف تصف صحتك العامة؟	[1] Very good جيدة جدا<br>[2] Good جيدة<br>[3] Fairمتوسطة<br>[4] Poor سيئة<br>[5] Very poor سيئة جدا
Section 3: Vaccination<br>القسم الثالث: التطعيم		
10	Which of the following vaccines do you know about are available in Egypt? (You can choose more than one answer. If you don't know which vaccine is available, you can leave it blank)	[1] Oxford/AstraZeneca اوكسفورد<br>[2] Moderna موديرنا<br>[3] Pfizer-BioNTech فايزر<br>[4] Sinovac (CoronaVac) سينوفاك<br>[5] Sinopharm (Verocell) سينوفارم<br>[6] Sputnik V سبوتنيك

	هل تعرف أيا من اللقاحات التالية المتاحة في مصر؟<br>(بامكانك اختيار أكثر من إجابة)	[7] Others غيرهم<br>[8] Dont know any of them لا أعرف أي منهم
11	Vaccine is legally mandatory?<br>هل لقاح الكورونا قانونا اجباري؟ أو<br>هل يلزمنا القانون بأخذ لقاح الكورونا؟	[1] Mandatory اجباري<br>[2] Voluntary اختياري<br>[3] Don't know لا أعلم
12	I agree that vaccines are harmful أتفق ان التطعيمات ضارة	[1] Not at all لا بالمرة<br>[2] No لا<br>[3] Neutral محايد<br>[4 [Yes نعم<br>[5] Yes completely نعم بالكلية
13	Vaccines can lead to death التطعيمات يمكن أن تؤدي للوفاة	[1] Yes نعم<br>[2] No لا<br>[3] Do not know لا أعلم
14	Rate your confidence in using local manufactured COVID-19 vaccine<br>ما مدى ثقتك في لقاح الكورونا المصنع محليا أو المصنوع في بلدك؟	[1] Completely not confidentلا أثق فيه على الاطلاق<br>[2] Not confidentلا أثق فيه<br>[3] Confidentأثق فيه<br>[4] Completely confidentأثق فيه تماما
15	Rate your confidence in using foreign manufactured (imported) COVID-19 vaccine<br>ما مدى ثقتك في لقاحات الكورونا المستوردة أو المصنعة في دول أجنبية؟	[1] Completely not confident غير واثق بالمرة<br>[2] Not confident غير واثق<br>[3] Confident واثق<br>[4] Completely confident واثق تماما
16	What percentage of persons in participants' circle are vaccinated<br>أي نسبة من الأشخاص في دائرتك تلقوا التطعيم؟	[1] 0-25% من صفر الى 25%<br>[2] >25%-50% أكثر من 25% الى 50%<br>[3] >50%-75% أكثر من 50% الى 75%<br>[4] >75%-100% أكثر من 75% الى 100%
17	If the booster dose of COVID-19 vaccine is available, would you take it?<br>اذا توفرت الجرعة المنشطة من لقاح الكورونا, هل ستتلقاها؟	[1] Definitely no بالتأكيد لا<br>[2] Probably no احتمال لا<br>[3] Possibly yes احتمال نعم<br>[4] Definitely yes بالتأكيد نعم

Appendix 2

Supplementary Table 1: Estimated marginal effects of intention to use a COVID-19 vaccine booster among subgroups based on the logit model

Factors		Unadjusted estimated marginal effect (95% CI)	P value	Adjusted estimated marginal effect (95% CI)	P value
1. Age		25.0 (24.7, 25.3)	0.23	25.0 (24.7, 25.3)	0.23
2. Sex	Male	70.9 (66.1, 75.3)	0.85	49.1 (22.7, 76.0)	0.50
	Female	70.4 (67.0, 73.5)		52.0 (24.9, 77.9)	
3. University student	Yes	67.9 (63.0, 72.5)	0.17	56.8 (28.7, 81.1)	0.04
	No	71.9 (68.6, 74.9)		44.2 (19.0, 72.8)	
4. Place of residence	Greater Cairo area	70.7 (62.9, 77.5)	0.16	55.7 (26.3, 81.7)	0.004
	Alexandria area	74.0 (69.2, 78.2)		54.7 (26.7, 80.0)	
	Delta area	67.2 (58.2, 75.2)		43.7 (18.3, 72.9)	
	Suez Canal area	73.7 (66.9, 79.5)		59.4 (29.9, 83.3)	
	Upper Egypt	66.0 (60.7, 71.0)		39.3 (16.2, 68.3)	
5. Chronic disease	Yes	70.5 (67.7, 73.2)	0.95	52.6 (25.7, 78.1)	0.67
	No	70.9 (57.7, 81.3)		48.5 (20.4, 77.6)	
6. COVID-19 infection	Yes	72.6 (69.2, 75.8)	0.049	53.0 (25.7, 78.6)	0.26
	No	67.1 (62.4, 71.4)		48.1 (22.0, 75.3)	
7. Perceived health	Very bad	50.0 (5.9, 94.1)	0.88	59.7 (0.07, 99.7)	0.53
	Bad	64.3 (37.6, 84.3)		34.1 (11.6, 67.1)	
	Average	69.1 (61.6, 75.7)		55.7 (40.6, 69.8)	
	Good	71.6 (67.7, 75.2)		54.5 (41.0, 67.4)	
	Very good	69.9 (65.2, 74.3)		49.0 (35.6, 62.6)	
8. Awareness of types of COVID-19 vaccines in Egypt		-	0.91	-	0.49
	Mandatory	68.2 (64.7, 71.4)	0..047	52.5 (25.4, 78.1)	0.63

9. Vaccines are mandated	Elective	76.2 (70.3, 81.2)		52.2 (24.6, 78.5)	
	Do not know	73.2 (66.0, 79.4)		46.9 (20.5, 75.2)	
10. Vaccines lead to death	Yes	68.9 (64.5, 73.0)	< 0.001	50.3 (23.4, 77.0)	0.76
	No	77.9 (74.1, 81.3)		52.7 (25.2, 78.6)	
	Do not know	54.4 (47.1, 61.5)		48.6 (22.0, 76.0)	
11. Vaccines are harmful	Not at all	90.1 (80.7, 95.2)	< 0.001	76.9 (44.5, 93.2)	< 0.001
	No	86.6 (82.8, 89.6)		67.2 (38.0, 87.3)	
	Neutral	65.8 (61.7, 69.8)		48.7 (22.4, 75.7)	
	Yes	36.5 (27.0, 47.2)		29.8 (10.6, 60.3)	
	Yes, completely	22.9 (13.2, 36.8)		28.8 (9.3, 61.6)	
12. Confidence in locally manufactured vaccines	Not at all	37.3 (30.2, 45.1)	< 0.001	33.8 (13.2, 63.1)	< 0.001
	No	65.3 (60.8, 69.4)		57.2 (29.0, 81.5)	
	Yes	87.0 (83.6, 89.8)		68.6 (39.9, 87.8)	
	Yes, completely	80.6 (64.5, 90.4)		42.2 (13.9, 76.8)	
13. Confidence in foreign manufactured vaccines	Not at all	20.5 (13.0, 30.9)	< 0.001	24.9 (8.2, 55.3)	< 0.001
	No	49.7 (43.9, 55.4)		35.6 (14.1, 65.2)	
	Yes	82.8 (79.7, 85.5)		66.2 (37.4, 86.6)	
	Yes, completely	89.4 (81.9, 94.0)		75.1 (44.4, 91.9)	
14. Percent vaccinated	0-25%	51.3 (40.2, 62.3)	< 0.001	41.1 (16.4, 71.1)	0.18
	>25%-50%	69.6 (62.7, 75.7)		57.2 (28.1, 82.0)	
	>50%-75%	68.6 (64.0, 72.8)		49.3 (22.8, 76.3)	
	>75%-100%	76.3 (72.0, 80.1)		54.6 (26.7, 79.9)	
15. Time in days the survey was open		-	0.95	-	0.99

CI: confidence interval
